# Focal hemolymphangioma of the rectum: A case report and literature review

**DOI:** 10.1097/MD.0000000000036666

**Published:** 2023-12-22

**Authors:** Wei Li, Binghu Jiang, Lifeng Zhou, Wenhua Liao

**Affiliations:** a Department of Radiology, Nanchong Central Hospital, North Sichuan Medical College, Nanchong, China; b Department of Gastroenterology, Nanchong Central Hospital, North Sichuan Medical College, Nanchong, China; c Department of Pathology, Nanchong Central Hospital, North Sichuan Medical College, Nanchong, China.

**Keywords:** case report, hemolymphangioma, radiology, rectum

## Abstract

**Rationale:**

Gastrointestinal hemolymphangiomas are very rare, especially in the rectum. Only 3 cases of rectal hemolymphangioma, all of which are diffuse lesions, have been reported in the English literature on PubMed. Our case is the first focal lesion of the rectum. It is important for radiologists to correctly identify the imaging features of rectal hemolymphangiomas.

**Patient concerns:**

A 51-year-old woman visited our hospital because of intermittent constipation for 3 years.

**Diagnoses:**

Colonoscopy revealed a prominent lesion on the left anterior wall of the lower rectum. Transvaginal color Doppler ultrasonography showed that the posterior vaginal wall area had a mixed-echo mass, and blood flow signals of the dots and stripes could be seen. Pelvic magnetic resonance imaging showed that the cystic space-occupying lesion in the region between the left anterior wall of the lower rectum and the posterior wall of the middle and lower vagina had a clear boundary.

**Interventions:**

The patient underwent surgery to remove the rectal lesions. The surgical specimen was finally diagnosed as local hemolymphangioma by pathological analysis.

**Lessons:**

Localized hemolymphangioma of the rectum is very rare, and imaging examination is essential for the diagnosis and evaluation of the extent of lesion invasion.

## 1. Introduction

Hemolymphangioma is a rare type of lymphatic malformation that involves a mixture of blood and lymphatic vessels. It often occurs in infants and young children, and is mostly located on the body surface.^[1]^ The incidence of hemolymphangioma in adults is extremely low, and there are few reports in the literature. At present, the occurrence sites reported in the literature are mostly the head and neck, and rarely the spleen, pancreas, retroperitoneum, esophagus, stomach, small intestine, colon, and rectum.^[2]^ However, there are only 3 cases of hemolymphangioma located in the rectum reported in PubMed,^[3–5]^ and all of which are diffuse lesions. This study is the first to report a case of localized hemolymphangioma in the lower segment of the rectum. Simultaneously, the literature on rectal hemolymphangioma was reviewed, and its clinical and imaging characteristics were summarized. In accordance with the CARE reporting checklist, we present the following case.

## 2. Case presentation

A 51-year-old female complained with intermittent constipation without obvious inducement 3 years ago, mainly with difficulty in defecation, and complained that the stool was not formed. The above symptoms occurred about once a year, and it could be relieved after about 1 week each time. The rest were not special, and the patient was not treated. One day ago, the patient suffered from the above symptoms again, so she went to the anorectal surgery department of Yuechi County People Hospital for treatment. During digital rectal examination, a 40*30 mm hard mass on the anterior wall of the rectum was found, with clear boundary, poor mobility and no tenderness. It was suggested that the patient should be further diagnosed and treated.

The patient visited the gynecology department of our hospital for various reasons. Physical examination of the gynecology department showed that a tumor could be palpable in the posterior vaginal wall and the anterior rectal wall, and the patient was recommended to be hospitalized. The patient underwent laparoscopic surgery for “ovarian chocolate cyst” 7 years ago. Relevant examinations were performed after admission, and routine blood, urine, stool, biochemical complete, and coagulation functions were normal, while carcinoembryonic antigen, carbohydrate antigen 125, 19-9, 72-4 were normal.

**Figure 1.**
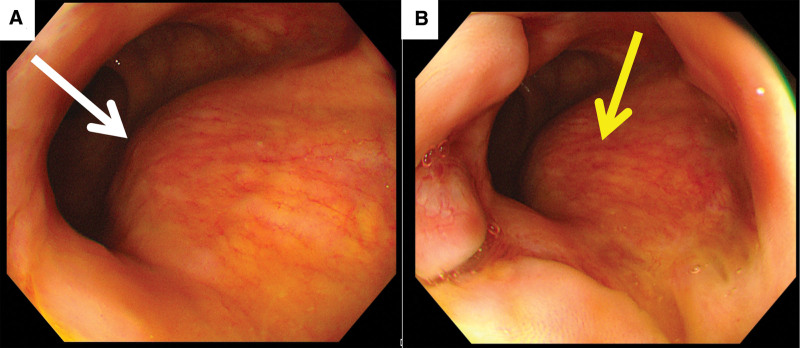
Colonoscopy. Submucosal protuberant lesion (white arrow) in the left anterior wall of the lower rectum. The intestinal mucosa of this segment is smooth and the vascular texture is normal. No active bleeding, edema, erosion, ulcer and other lesions are seen (yellow arrow).

**Figure 2.**
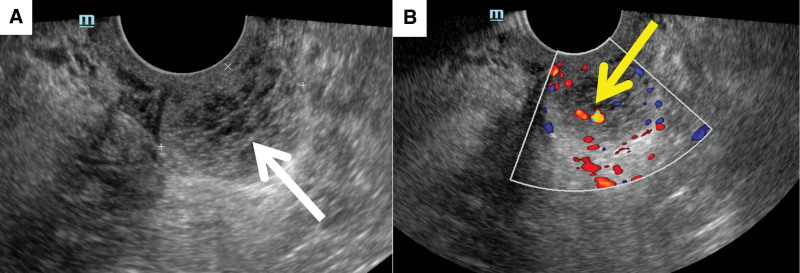
Transvaginal color Doppler ultrasonography. (A) A systic-solid mixed echo mass (white arrow) in the posterior vaginal wall area, with a size of about 30 × 23 × 26 mm, with regular shape and clear boundary. (B) Color Doppler ultrasound showing a dotted and streaky blood flow signal (yellow arrow) at its internal lesion.

**Figure 3.**
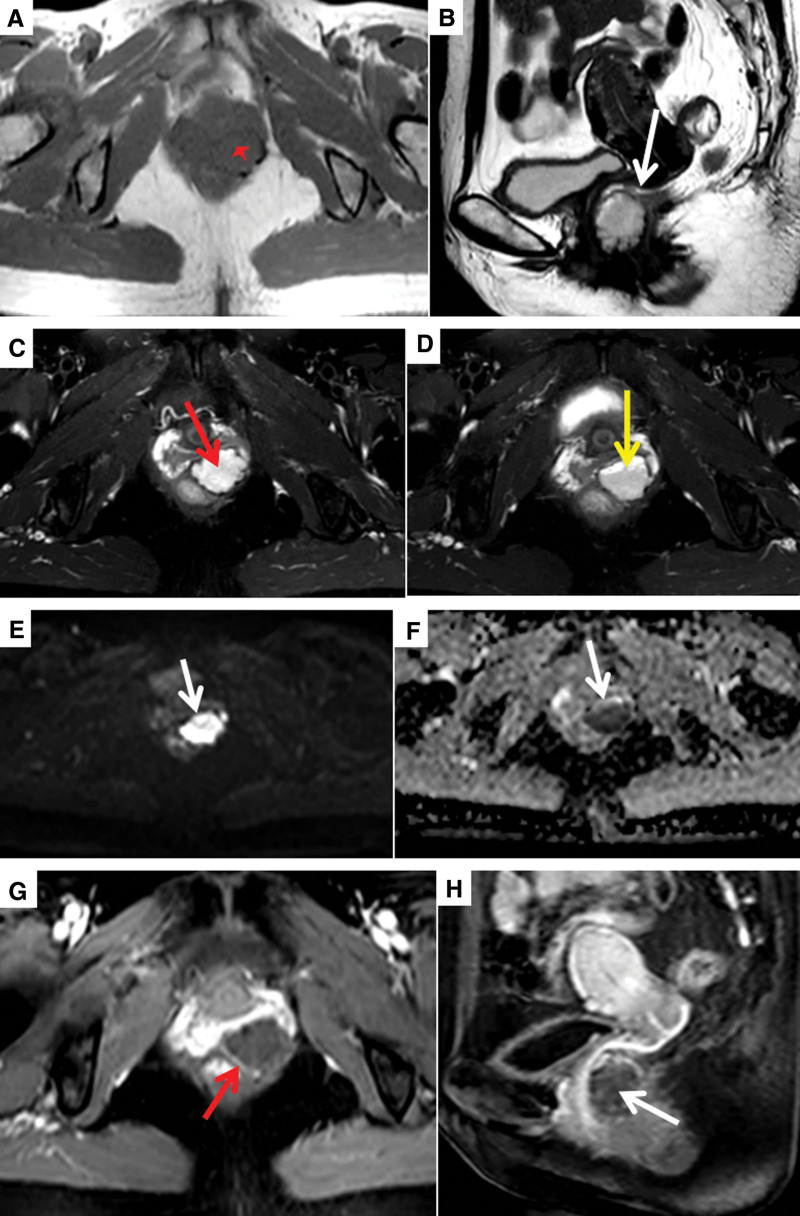
Pelvic MRI examination. (A, Axial T1WI) and (B, Sagittal T2WI), A lobulated mass (red star) can be seen in the gap between the posterior wall of the middle and lower segment of the vagina and the left anterior wall of the rectum, with a clear boundary and a size of about 30 × 22 × 28 mm, which is connected with the seromuscular layer of the left anterior wall of the rectum at an obtuse angle (white arrow), and the adjacent posterior vaginal wall is compressed and displaced forward, showing a slightly low signal on T1WI and an obvious high signal on T2WI. (C and D, Axial T2WI fat suppression sequence) shows mixed hyperintensity, with multiple fine separations (red arrow) and layered changes (liquid-liquid level, yellow arrow). (E and F) The diffusion of rectal lesions is significantly restricted (DWI high signal, ADC mixed low signal, white arrow). (G and H) After enhance, the cyst wall at the edge of the lesion showing moderate enhancement (red arrow), the septum showing mild enhancement (white arrow), and the cystic component showing no enhancement. ADC = appearant difussion coefficient, DWI = diffusion weighted imaging, MRI = magnetic resonance imaging, T1WI = T1 weighted imaging, T2WI = T2 weighted imaging.

Colonoscopy revealed that the left anterior wall of the lower segment of the rectum was a protuberant lesion. The intestinal mucosa of this segment was smooth; the vascular texture was normal; and no bleeding, erosion, ulcers, or other manifestations were observed (Fig. 1). The patient transvaginal color Doppler ultrasound examination showed that the posterior vaginal wall area was a cystic-solid mass with mixed echo, with a size of about 30 × 23 × 26 mm, with regular shape and clear boundary, and dotted and strip blood flow signals can be seen in it (Fig. 2). Subsequent Pelvic magnetic resonance imaging (MRI) revealed that the cystic space-occupying lesion in the region between the left anterior wall of the lower rectum and the posterior wall of the middle and lower vagina had a clear boundary that was connected to the seromuscular layer of the left anterior wall of the rectum at an obtuse angle. The posterior wall of the vagina was compressed and shifted forward. There was a slightly low signal on T1 weighted imaging, an obvious high signal on T2 weighted imaging (T2WI), and a mixed high signal on T2WI fat-suppression sequence. There were multiple small septations and layering changes (liquid-level display) and diffusion was significantly restricted. After enhancement, the lesion edge was moderately enhanced, septation was mildly enhanced, and the cystic components were not enhanced (Fig. 3).

The patient had a space-occupying lesion in the vaginal rectal septum, the nature of which was unknown, and underwent surgery. Intraoperative findings: There was a hard mass approximately 28 × 30 mm in size between the lower vaginal segment and rectum, the boundary was clear, and the motion was poor. The intraoperative freezing pathology suggested a benign lesion. The muscularis of the anterior wall of the rectum was partially invaded by the tumor. The muscularis was thin after the tumor was stripped. Absorbable sutures were used for the thin muscles. The rectal mucosa remained intact after anal examination.

**Figure 4.**
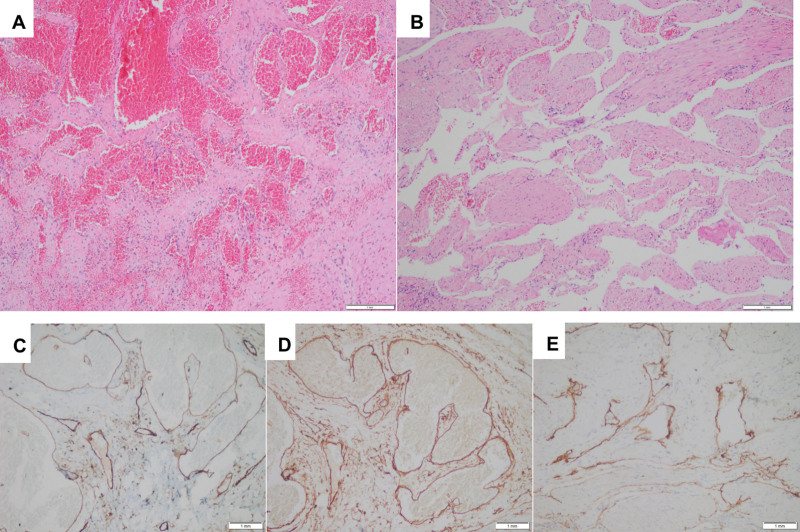
Pathological examination. (A and B) HE staining × 100 microscopic examination shows that there are multiple dilated blood vessels and lymphatic vessels with different sizes, and some of them are dilated into cysts, consistent with hemolymphangioma. Immunohistochemistry shows that tumor cells express CD31and CD34 (vascular +) (C and D), D2-40 (lymphatic +) (E). The final pathological diagnosis is hemolymphangioma of rectal seromuscular layer.

Postoperative pathology revealed the capsule wall of the vaginal rectal septum lesion and a piece of grayish red and grayish brown soft tissue, approximately 25 × 20 × 8 mm. Microscopic examination revealed multiple dilated lymphatic vessels and vascular structures, consistent with a hemolymphangioma (Fig. 4A and B). Immunohistochemistry showed that the tumor cells expressed CD34, CD31 (vascular +) (Fig. 4C and D), and D2-40 (lymphatic +) (Fig. 4E). The final pathological diagnosis was rectal hemolymphangioma. The patient was followed up for 3 months postoperatively without signs of recurrence.

## 3. Discussion

Lymphangiomas are usually divided into 4 types, capillary lymphangiomas, cavernous lymphangiomas, cystic lymphangiomas, and hemolymphangiomas.^[6]^ Hemolymphangioma is rare in the clinic, mostly congenital, and its specific formation mechanism is not clear. This may be caused by congenital malformations in the development of vasculature. Tumor formation is caused by the obstruction of veno-lymphatic communication between the systemic circulation and dysembryoplastic vascular tissue.^[7]^ Trauma and surgery may lead to injury of blood vessels and lymphatic vessels, resulting in obstruction of reflux, thus forming hemolymphangioma; however, it is extremely rare.^[8]^ Although this patient had previously undergone laparoscopic surgery for “ovarian chocolate cyst,” but it could not affect the rectal vasculature.

Intestinal hemolymphangiomas are rare. The common clinical manifestations are painless bloody stool, anemia, abdominal distension, and intestinal obstruction etc^.[9]^ The lesion in this case was located in the seromuscular layer; therefore, there were no symptoms or signs of bloody stool and anemia. It is often difficult to distinguish it from other space-occupying diseases of the rectum, so there are huge difficulties in preoperative and intraoperative diagnosis. Preoperative diagnosis mainly relies on colonoscopy and imaging examination^[4,7,10–13]^: Colonoscopy, the lesions of mucosa and submucosa can be seen as blue-purple and dark-red nodules on the surface of the intestinal mucosa and active bleeding, erosion, ulcers, congestion, and edema of the intestinal mucosa of the lesion. Endoluminal or endoscopic ultrasound can reveal thickening of the intestinal wall with multiple cystic hypoechogenicity. Color Doppler imaging revealed that the internal blood flow was relatively rich. There may be a large number of tortuous and dilated venous vascular plexuses around the intestine. Part of the intestinal wall has multiple punctate hyperechogenicity with acoustic shadows owing to the presence of phleboliths. The appearance of calcified phleboliths in the lesions on plain computed tomography (CT) has certain characteristics. The enhancement performance of CT depends mainly on the proportion of blood vessels in the tumor. For those with a high proportion of blood vessels, significant enhancement can be seen on enhanced scan, and the enhancement in the venous phase and delayed phase is more obvious; for those with a low proportion of blood vessels, the enhancement is not obvious. On MRI, the cystic fluid signal is also related to the ratio of blood vessels to lymphatic vessels in the tumor. T1 weighted imaging has a low signal, and T2WI has a high signal with multiple septations. The lesion with hemorrhage can be seen at the liquid-liquid level. Diffusion weighted imaging shows a high signal, the appearant difussion coefficient value is reduced, and its enhancement performance is similar to that of CT. After the MRI enhancement scan, the tumor margin with moderate enhancement and multiple septations with mild enhancement in the lesion could be seen in the arterial phase and progressive enhancement in the venous phase, while the delay period was more obvious. The final diagnosis of hemolymphangioma mainly depends on the postoperative pathological examination. Intraoperative gross anatomy and frozen biopsies are important for diagnosis. Hemolymphangiomas usually have soft texture, clear boundaries, and multicystic changes. The cystic fluid is the blood and lymph.^[10]^ The dissection of the hemolymphangioma specimen in this case showed that the cut surface was grayish red and grayish brown, and was covered with multiple small cystic cavities. A large number of dilated blood vessels and lymphatic vessels can be seen under the microscope, and immunohistochemistry revealed that both vascular endothelial cells and lymphatic endothelial cells express CD31 and CD34. D2-40 is only expressed in lymphangiomas and some malignant vascular tumors, which is not difficult to diagnose in combination with the above indicators.^[11]^ The tumor cells expressed CD31 and CD34 (vascular +) and D2-40 (lymphatic +), and the pathological diagnosis of hemolymphangioma was definite.

At present, only 3 cases of rectal hemolymphangioma have been reported in the literature. The clinical manifestations and imaging characteristics of the patients differed.^[3–5]^ There were 2 males and 1 female, both of whom had diffuse lesions of the rectal wall. One case involved the sigmoid colon and presacral space. To the best of our knowledge, this is the first report of a localized hemolymphangioma of the rectal seromuscular layer. This type is characterized by small lesions and late and mild clinical symptoms and signs. However, enteroscopy and endoscopic ultrasonography can reveal mucosal bleeding points and intestinal wall layers of the lesion origin. Based on a review of the known literature and this case, we believe that the MRI findings of localized hemolymphangioma of the rectum still have some characteristics. For example, the lesion is small, well defined, and confined to the intestinal wall. Second, the lesion was mainly cystic with multiple septations. T2WI shows obvious hyperintensities, and fluid levels can be observed when combined with hemorrhage. Third, diffusion weighted imaging was significantly diffusion-limited, and the appearant difussion coefficient value decreased. Fourth, the cystic wall and septum were slightly or moderately enhanced after enhancement, whereas the cystic components were not enhanced.

Because the nature of preoperative diagnosis of rectal hemolymphangioma is not clear, there may be risks of massive bleeding, secondary infection and intestinal obstruction. The treatment of hemolymphangioma is mainly surgical resection.^[7]^ Treatment options, such as surgery, endoscopic surgery (ligation, cauterization, intratumoral injection of sclerosing agents), are recommended, and drugs can be used for small lesions.^[13,14]^ Because rectal hemolymphangioma is a benign tumor, anus-preserving surgery should be used as much as possible; therefore, anal sphincter function should be preserved to improve the quality of life of patients while ensuring a negative resection margin. Thus, it is important to determine the location of the lesion, extent of involvement, and distance to the anal margin before surgery. Diffuse lesions usually have a large size and a wide range of infiltration, easily involving the sigmoid colon and anal canal, peripheral mesentery, and pelvic organs, and the clinical symptoms appear early and serious.^[3,4]^ Imaging examination can clarify the scope of the lesion and the affected organs, as well as the distance from the lesion to the anal margin. For patients with a large range of lesions, the operation may be difficult and the requirements for the operator are high. Lesions should be completely removed during surgery. The prognosis of hemolymphangioma is good. The recurrence rate after complete resection is low, ranging from 10% to 27%, whereas the recurrence rate after partial resection ranges from 50% to 100%.^[15]^ Thus regular follow-up is required after surgery. The patient was followed-up for 3 months after the operation, and the prognosis was good, without signs of recurrence.

Although the disease is benign, it is characterized by invasive growth and is easily misdiagnosed as a malignant tumor.^[16,17]^ It should be differentiated from the following tumors: Rectal gastrointestinal stromal tumors. When the tumor is small, the signal is uniform, and the lesions on the enhanced scan show uniform moderate or obvious enhancement, especially in the portal venous phase; when the tumor is large, necrosis and cystic changes may occur, and the signal is uneven. The air-liquid plane can be seen after communicating with the intestinal cavity. An enhancement scan shows the blood supply artery.^[18]^ Rectal cancer. Most show circumferentially infiltrative growth with irregular morphology, resulting in luminal stenosis and intestinal obstruction, and often mesentery invasion and regional lymph node metastasis.^[19]^ Rectal mucosa-associated lymphoid tissue lymphomas. Most of these are annular thickening of the intestinal wall or polypoid protrusion into the intestinal lumen. The bowel wall was soft and the internal signal was uniform. The intestinal lumen can undergo an aneurysm-like expansion. Enlarged lymph nodes were observed in the medial and proximal lymphatic drainage areas of the tumor.^[20]^ Rectal NETs. Rectal NETs are mostly grade 1, mild enhancement in the arterial phase and obvious enhancement in the venous phase. High-grade tumors are generally large in size, infiltrative growth, and relatively more prone to cystic degeneration and necrosis, arterial phase enhancement, and pelvic lymphadenopathy, suggesting that high-grade ENTs may be present.^[21]^

## 4. Conclusion

This paper reports a case of hemolymphangioma of the rectal seromuscular layer. Focal hemolymphangioma of the rectum shows no special symptoms or signs. Owing to the small volume and deep location of the tumor in the early stage, which is difficult to show by conventional endoscopy, it is easy to miss and misdiagnose clinically. Therefore, although the incidence of rectal haemolymphangioma is very low, the possibility of this disease should be considered in patients with intestinal symptoms accompanied by bleeding and anemia. Imaging is essential for the diagnosis and evaluation of the extent of lesion invasion. Focal hemolymphangioma of the rectum is very rare. Clinicians and radiologists still have a serious lack of knowledge of it. It is necessary to improve the understanding of it in order to avoid misdiagnosis and mistreatment.

## Author contributions

**Conceptualization:** Wei Li, Binghu Jiang, Lifeng Zhou, Wenhua Liao.

**Data curation:** Wei Li.

**Investigation:** Wei Li.

**Resources:** Wei Li, Lifeng Zhou, Wenhua Liao.

**Supervision:** Binghu Jiang.

**Validation:** Binghu Jiang.

**Writing – original draft:** Wei Li, Binghu Jiang.

**Writing – review & editing:** Wei Li, Binghu Jiang.
